# A New Electrically Conducting Metal–Organic Framework Featuring U-Shaped *cis*-Dipyridyl Tetrathiafulvalene Ligands

**DOI:** 10.3389/fchem.2021.726544

**Published:** 2021-10-01

**Authors:** Monica A. Gordillo, Paola A. Benavides, Kaybriana Spalding, Sourav Saha

**Affiliations:** Department of Chemistry, Clemson University, Clemson, SC, United States

**Keywords:** metal-organic frameworks, redox-active, tetrathiafulvalene, electrical conductivity, iodine

## Abstract

A new electrically conducting 3D metal-organic framework (MOF) with a unique architecture was synthesized using 1,2,4,5-tetrakis-(4-carboxyphenyl)benzene (TCPB) a redox-active *cis*-dipyridyl-tetrathiafulvalene (*Z*-DPTTF) ligand. While TCPB formed Zn_2_(COO)_4_ secondary building units (SBUs), instead of connecting the Zn_2_-paddlewheel SBUs located in different planes and forming a traditional pillared paddlewheel MOF, the U-shaped *Z*-DPTTF ligands bridged the neighboring SBUs formed by the same TCPB ligand like a sine-curve along the b axis that created a new *sine*-MOF architecture. The pristine *sine*-MOF displayed an intrinsic electrical conductivity of 1 × 10^−8^ S/m, which surged to 5 × 10^−7^ S/m after I_2_ doping due to partial oxidation of electron rich *Z*-DPTTF ligands that raised the charge-carrier concentration inside the framework. However, the conductivities of the pristine and I_2_-treated *sine*-MOFs were modest possibly because of large spatial distances between the ligands that prevented π-donor/acceptor charge-transfer interactions needed for effective through-space charge movement in 3D MOFs that lack through coordination-bond charge transport pathways.

## Introduction

Metal organic frameworks (MOFs) are versatile materials with diverse structures, composition, properties, and functions (Furukawa et al., 2013; Yuan et al., 2018). These characteristics of MOFs have attracted researchers because of their potential applications in catalysis (Liu et al., 2014; Dolgopolova and Shustova, 2016), guest separation (Li et al., 2012), storage (Sumida et al., 2012), and delivery, light harvesting (Zhang and Lin, 2014; Liu et al., 2015; So et al., 2015; Maza et al., 2016; Gordillo et al., 2019), ionic and electronic conduction (Horike et al., 2013; Ramaswamy et al., 2014; Stavila et al., 2014; Sun et al., 2016; Stassen et al., 2017), and sensing (D’Alessandro, 2016; Lustig et al., 2017; Khatun et al., 2019), among other advanced applications (Rice et al., 2020). Introducing redox-active ligands is a rather simple way to elicit multifunctionality in MOFs, a strategy that has been widely adopted in recent years (Ding et al., 2021). One of the most commonly used redox-active ligands is tetrathiafulvalene (TTF) (Segura and Martin, 2001; Canevet et al., 2009; Wang et al., 2017b), a sulfur containing electron-rich molecule that possesses two easily accessible redox states, i.e., TTF^•+^ radical cation and TTF^2+^ dication that has been widely employed as an electron donor in optoelectronic (Wang et al., 2017b), conductive (Narayan et al., 2012; Sun et al., 2016), and magnetic materials (Wang et al., 2017a). Equipped with two pyridyl groups on the TTF core, dipyridyl tetrathiafulvalene (DPTTF) ligand not only inherits the redox properties of parent TTF, but also becomes capable of coordinating metal ions. The *Z*-DPTTF ligand, however, exists in a mixture of *E* and *Z*-isomers, with the latter surprisingly being the major isomer. The *E* isomer adopts a nearly linear shape, whereas the *Z*-isomer adopts a U-shape, which is probably one of the reasons why this ligand has not been as extensively incorporated in MOFs (Yin et al., 2016; Sherman et al., 2020; Weng et al., 2020) as other TTF derivatives (Park et al., 2015; Wang et al., 2015; Su et al., 2017; Wang et al., 2017a; Leong et al., 2018; Wang et al., 2019). Herein, we report the synthesis of a new electrically conducting *sine*-MOF [Zn_2_ (DPTTF)TCPB•3DMA]_n_ featuring 1,2,4,5-tetrakis-(4-carboxyphenyl)benzene (TCPB) and *Z*-DPTTF where the former formed Zn_2_(COO)_4_ paddlewheel nodes while the latter connected the adjacent nodes formed by the same TCPB ligands via axial coordination in such a way that two U-shaped *Z*-DPTTF ligands axially coordinated to the same Zn_2_ paddlewheel node completed a *sine*-wave propagating along the b-axis. This new *sine*-MOF displayed a 50-fold increase in room temperature electrical conductivity from 1 × 10^−8^ to 5 × 10^−7^ S/m after I_2_ doping largely due to partial oxidation of the electron rich *Z*-DPTTF ligands, which enhanced the charge-carrier concentration.

## Experimental Section

### Materials

Reagents, starting materials, and solvents were purchased from Sigma-Aldrich, Acros Organic, TCI America and EMD Chemicals and used as received. *Z*-DPTTF ligand was prepared following a literature protocol (Han et al., 2007).

### Preparation of *sine*-MOF

To a solution of *Z*-DPTTF ligand (7.2 mg, 0.02 mmol) in DMAc (2 ml) placed in a screw-capped vial, a separately prepared solution of Zn(NO_3_)_2_•6H_2_O (11.9 mg, 0.04 mmol) and TCPB (11.2 mg, 0.02 mmol) in 2:1 DMAc/H_2_O mixture (1.5 ml) was added slowly. Once all these starting materials were fully dissolved upon gentle shaking, 1 M HNO_3_ ethanolic solution (20 µl) was added to it. The resulting mixture was then heated in an oven at 80°C for 24 h. The resulting dark-red crystals (48%) were used for single-crystal x-ray diffraction (SXRD) analysis and the corresponding evacuated powder was used for electrical and optical measurements. Elemental analysis: Calc. for Zn_2_C_52_H_37_O_10.5_S_4_N_2_: C 55.92, H 3.34, and S 11.48%. Found: C 56.01, H 3.44, and S 11.53%

### Preparation of I_2_ Doped *sine*-MOF

The dark-red colored evacuated *sine*-MOF powder was placed in a small open vial, which was then placed inside a larger screw-capped vial containing few I_2_ chips. The larger vial was capped tightly and sealed with parafilm tape to keep the *sine*-MOF crystals exposed to iodine vapor for 3 days, which caused the *sine*-MOF powder to turn black. The I_2_-treated *sine*-MOF vial was removed from the I_2_ chamber, left open overnight, and finally washed thoroughly with hexane until the washing solution became colorless indicating that the material was free of any physisorbed I_2_. Elemental analysis: Calc. for Zn_2_C_55_H_50_O_14_S_4_N_2_I_1.5_: C 46.77, H 3.57, S 9.08%, and I 13.48. Found: C 46.97, H 2.80, S 8.24, and I 13.44%.

## Results and Discussion

### Synthesis, Structural Characterization, and Thermogravimetric Analysis of *sine*-MOF

**FIGURE 1:**
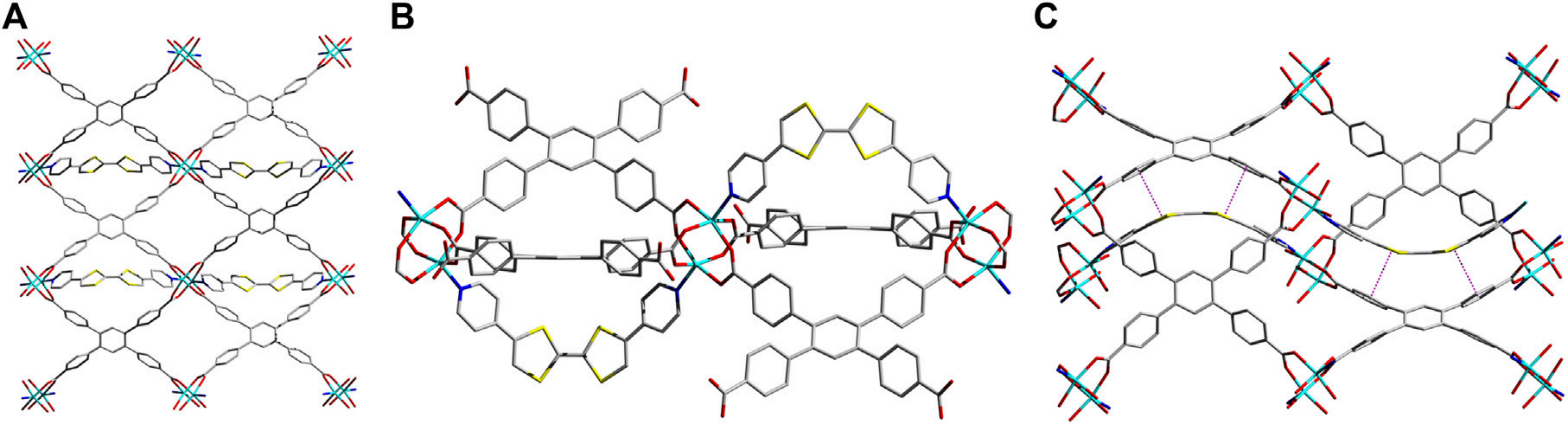
**(A)** Crystal structure of *sine*-MOF [Zn_2_ (DPTTF)TCPB•3DMA]_n_ viewed along the c axis. **(B)** The paddlewheel-like SBUs formed by the TCPB ligands are connected by axially coordinated U-shaped *Z*-DPTTF ligands extended along the b-axis. **(C)** A view of the sinusoidal thread formed by *Z*-DPTTF ligands by bridging adjacent SBU units along the b axis and the π-π interactions between the dithiolene rings of the TTF core and two benzoate moieties of the TCPB ligand with a centroid-to-centroid distance of 3.66 Å. Solvent molecules and H atoms are omitted for clarity. Atom legends: cyan, Zn(II); blue, N; red, O; yellow, S; and gray, C.

A solvothermal reaction between Zn_2_(NO_3_)_2_•6H_2_O, TCPB, and *Z*-DPTTF in a DMAc/H_2_O mixture at 80°C for 24 h yielded dark-red *sine*-MOF crystals. SXRD analysis revealed that *sine*-MOF [Zn_2_ (DPTTF)TCPB•3DMA]_n_ crystallized in an orthorhombic space group Pnma (Figures 1A,B and Supplementary Figure S1). The TCPB ligands formed Zn_2_(COO)_4_ paddlewheel nodes, but unlike typical pillared paddlewheel MOFs, they did not form 2D sheets of these nodes in *sine*-MOF. Instead, they formed a 3D framework thanks to a significant twist of TCPB ligand, which was evident from large dihedral angles (ca. 43–47°) between the central benzene ring and terminal benzoate rings. The axial sites of the Zn_2_(COO)_4_ paddlewheel nodes were occupied by *Z*-DPTTF ligands, although these dipyridyl ligands did not act as typical pillars found in pillared paddlewheel MOFs. Instead, each U-shaped *Z*-DPTTF ligand bridged two adjacent Zn_2_ nodes formed by two 1,3-benzoate groups of the same TCPB ligand. Each Zn_2_ paddlewheel node carried one U-shaped *Z*-DPTTF ligand at the top axial position and another at the bottom axial position, which then bridged two adjacent Zn_2_ nodes from the top and bottom axial positions, respectively. Thus, the consecutive Zn_2_ nodes located along the b-axis were connected by U-shaped *Z*-DPTTF ligands in an alternating top/bottom fashion that resembled a full sine-wave (Figure 1C), prompting us to label this new architecture as *sine*-MOF. The formation of this uncommon architecture was made possible by the bent geometry of *Z*-DPTTF ligands having an angle of 36° between the two dithiolene rings and a dihedral angle of 10° between two *cis*-pyridyl rings. The bent geometry and short central C=C bond length (1.34 Å) of *Z*-DPTTF indicated that they existed in the neutral form in pristine *sine*-MOF (Gao et al., 2014; Su et al., 2017). Due to an alternate up/down orientation of *Z*-DPTTF ligands, *sine*-MOF lacks intermolecular π–π and S•••S interactions between the TTF cores, but it enjoys π–π interaction between the dithiolene rings of *Z*-DPTTF and two benzoate rings of TCPB ligand that have a centroid-to-centroid distance of 3.66 Å (Supplementary Figure S2).

**FIGURE 2:**
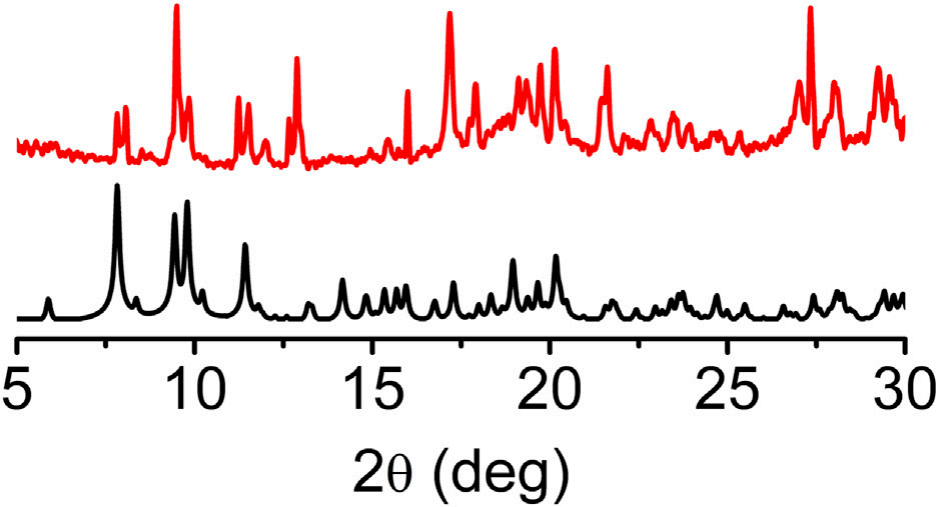
PXRD pattern of pristine *sine*-MOF showing retention of the crystallinity after activation.

The experimental powder X-ray pattern (PXRD) of pristine *sine*-MOF was consistent with the simulated one obtained from the SXRD analysis, which confirmed the phase purity and crystallinity of the evacuated bulk material (Figure 2). Iodine is a mild oxidant that is known to chemically oxidize TTF and other electron rich π-systems (Su et al., 2017; Gordillo et al., 2020). Exposure of pristine *sine*-MOF crystalline powder to I_2_-vapors afforded a black material that was washed thoroughly with hexanes until the wash solution became colorless indicating that no residual physisorbed I_2_ was left in the I_2_-treated *sine*-MOF. The PXRD pattern of the I_2_-treated *sine*-MOF matched with that of the pristine material, confirming the retention of its structural integrity and crystallinity (Supplementary Figure S3).

**FIGURE 3:**
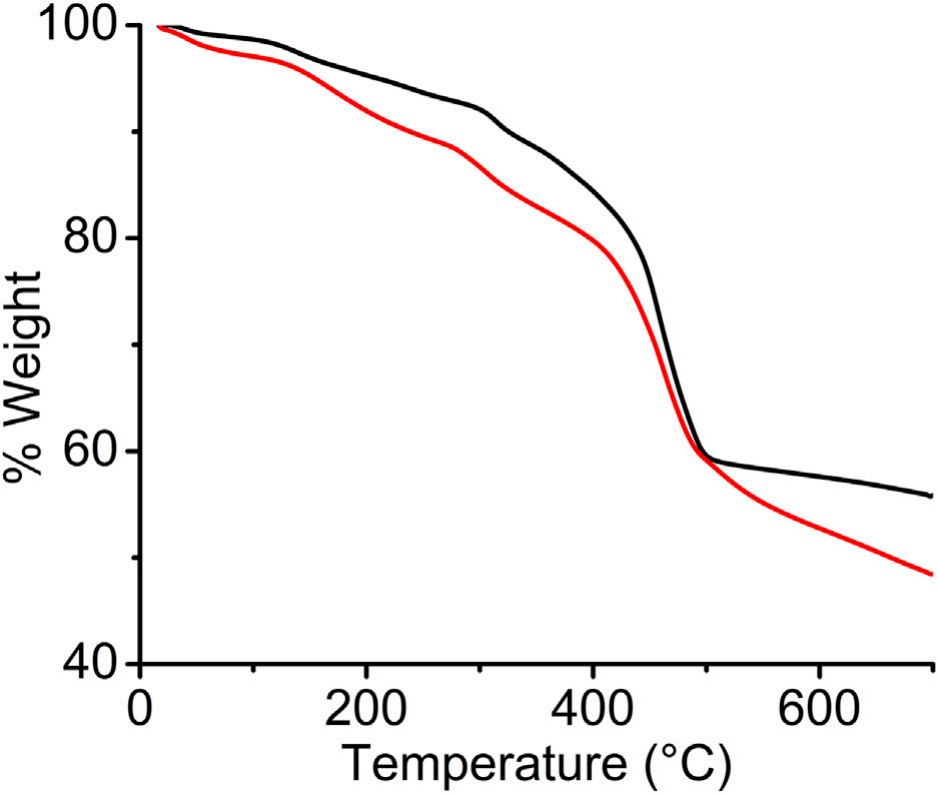
TGA profiles of pristine (black) and I_2_-treated (red) *wavy*-MOF.

Thermogravimetric analysis (TGA) was performed on vacuum-dried pristine and I_2_-treated *sine*-MOF samples in N_2_ atmosphere (Figure 3). The TGA profile of pristine *sine*-MOF revealed a gradual 10% weight loss until 300°C corresponding to the loss of solvent molecules, followed by a sharp 33% weight loss due to framework decomposition. The TGA profile of the I_2_-doped *sine*-MOF displayed an initial weight loss of 14% corresponding to the loss of MeOH and water molecules, followed by another significant weight loss step above 400°C that corresponded to framework decomposition.

### Optical and Electrochemical Properties of *sine*-MOF

**FIGURE 4:**
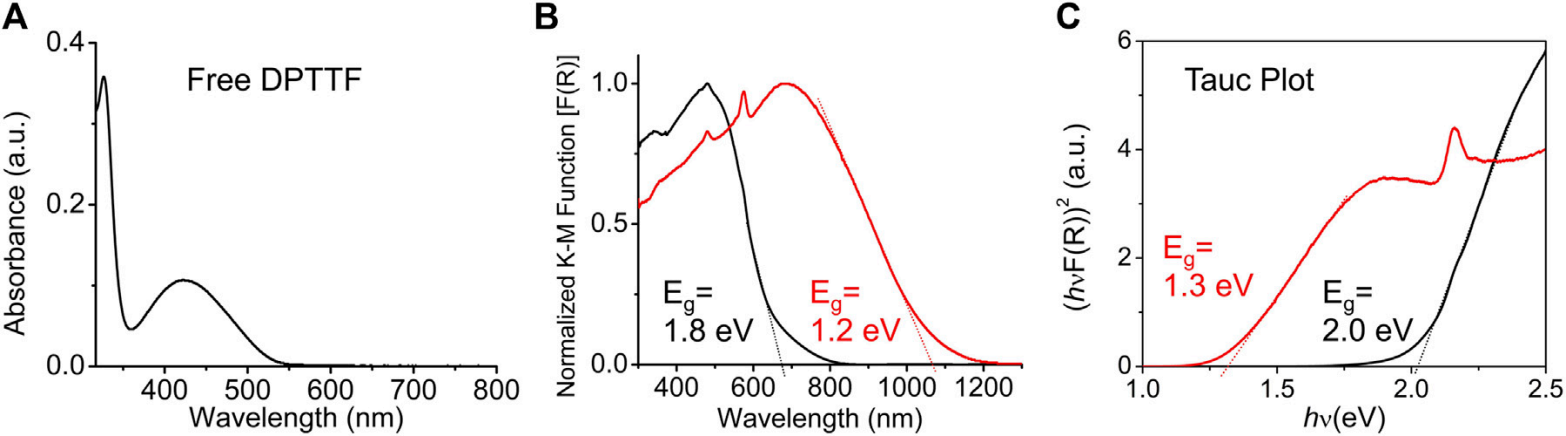
**(A)** UV-vis absorption spectrum of *Z*-DPTTF ligand in DMF. **(B)** Diffusion reflectance spectra of pristine (black) and I_2_-treated (red) *sine*-MOF. **(C)** The Tauc plot of pristine (black) and I_2_-treated (red) *sine*-MOFs.

The diffuse reflectance spectra (DRS) of pristine and I_2_-doped *sine*-MOFs were measured. Pristine *sine*-MOF displayed a broad band centered on 480 nm, which was ca. 60 nm red-shifted with respect to the UV-vis absorption spectrum (λ_max_) of *Z*-DPTTF recorded in DMF (Figures 4A,B). From the onset of the λ_max_ peak of the *Z*-DPTTF ligand its optical bandgap of 2.3 eV was calculated. The optical bandgaps of pristine and I_2_-treated *sine*-MOFs (*E*_g_ = 1.8 and 1.2 eV, respectively) (Figure 4B) were narrower than that of the free ligand, probably because of π–π and π-donor/acceptor interactions between the *Z*-DPTTF and TCPB ligands in pristine and I_2_-treated *wavy* MOFs, respectively. The results from the corresponding Tauc plot (Figure 4C) were in good agreement with those determined from DRS and revealed ∼0.6–0.7 eV narrower bandgap for the I_2_-doped *sine*-MOF with respect to the pristine MOF. The narrower bandgap of I_2_-treated *sine*-MOF is likely due to a partial oxidation of the *Z*-DPTTF ligands to *Z*-DPTTF^•+^ radical cations within the framework.

**FIGURE 5:**
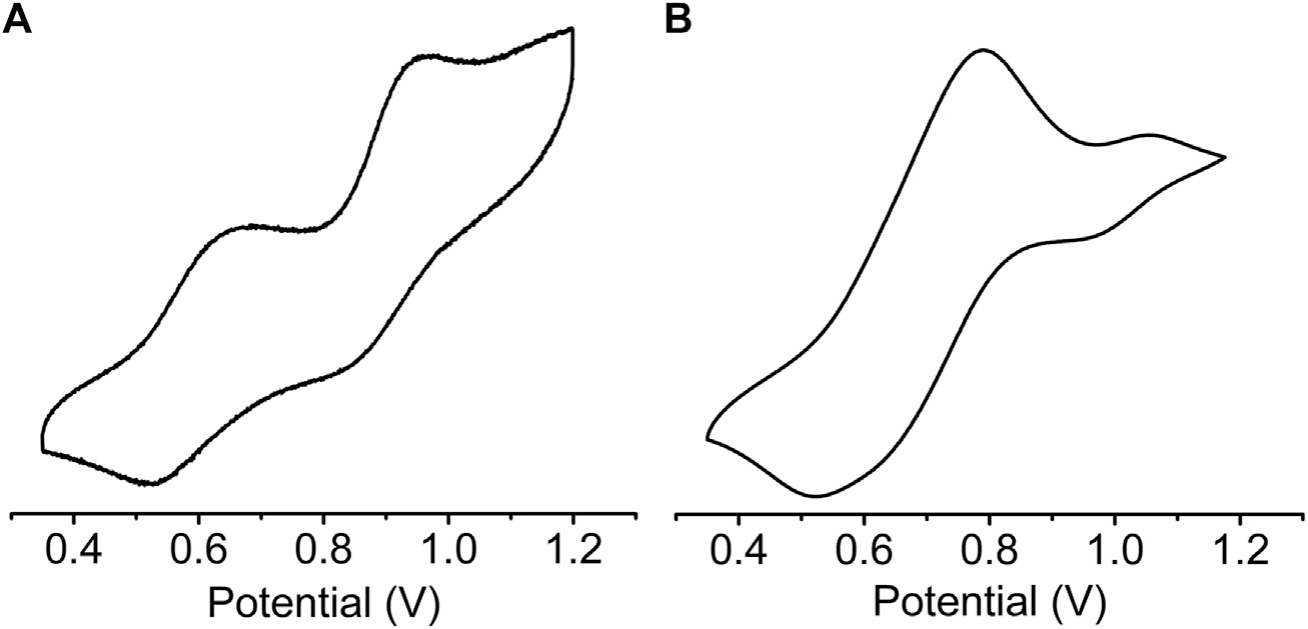
Cyclic Voltammograms of **(A)** pristine and **(B)** I_2_-treated *sine*-MOF (vs Ag/AgCl, 0.1 M Bu_4_NPF_6_ in MeCN).

Solid state cyclic voltammetry (CV) (Figure 5) and square wave voltammetry (SWV) (Supplementary Figure S4) were used to investigate the redox properties of *sine*-MOF. TTF compounds are known to display two reversible one electron oxidation steps corresponding to TTF^•+^ and TTF^2+^ formation. The CV of pristine *sine*-MOF displayed two quasi-reversible oxidation processes (Figure 5A) with anodic peaks at 0.68 and 0.96 V (vs Ag/AgCl, 0.1 M Bu_4_NPF_6_ in MeCN) corresponding to stepwise one-electron oxidation of *Z*-DPTTF to *Z*-DPTTF^•+^ and *Z*-DPTTF^2+^. The anodic peaks of I_2_-doped wavy-MOF (Figure 5B) appeared toward more positive potentials at 0.79 and 1.05 V suggesting that such I_2_-mediated partially oxidized framework was more difficult to oxidize electrochemically than pristine *sine*-MOF.

**FIGURE 6:**
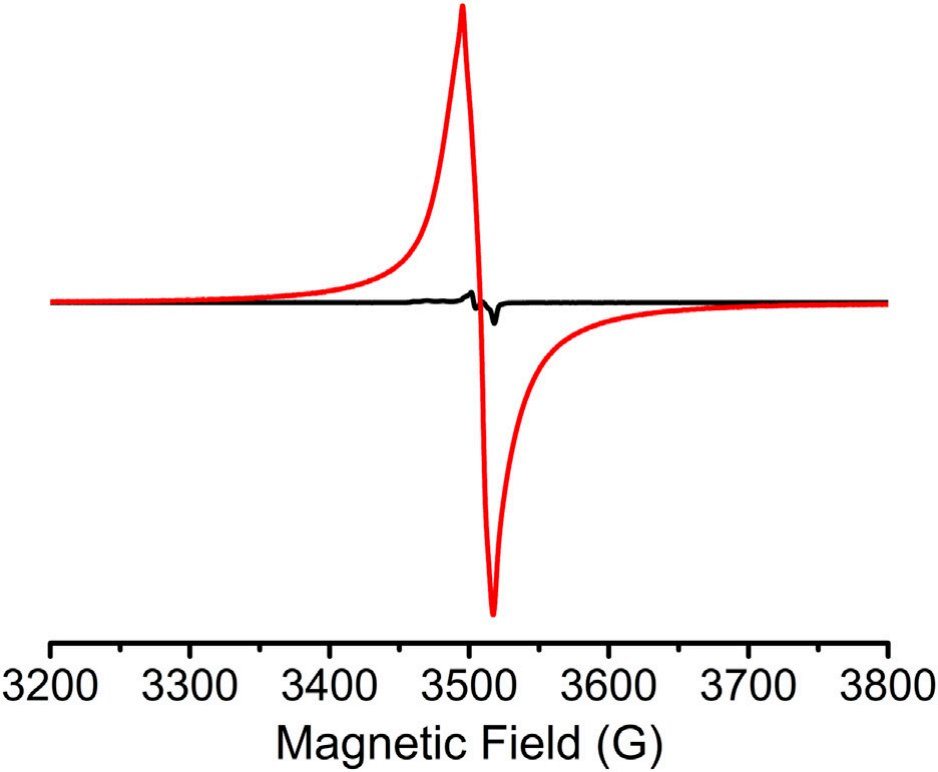
Solid-state EPR spectra of pristine (black) and I_2_-treated (red) *sine*-MOF.

Solid-state electron paramagnetic resonance (EPR) confirmed the presence of *Z*-DPTTF^•+^ radical cations within the *sine*-MOF. A weak EPR signal (Figure 6) was present in pristine *sine*-MOF indicating that most of the *Z*-DPTTF ligands were in the neutral state and that a small percentage may have been oxidized by air as has been previously reported for other TTF-based MOFs. (Narayan et al., 2012; Park et al., 2015; Su et al., 2017; Wang et al., 2017a; Leong et al., 2018). In contrast, a strong EPR signal (g ≈ 2.006) was observed (Figure 6) for I_2_-treated *sine*-MOF indicating that a significant population of *Z*-DPTTF ligands were oxidized to paramagnetic *Z*-DPTTF^•+^ radical cations. The elemental analysis data of I_2_-treated *sine*-MOF (*vide supra*) corresponded to an empirical formula of Zn_2_C_55_H_50_O_14_S_4_N_2_I_1.5_. Based on the I/S ratio, we estimated that there was roughly one I_3_^−^ anion for two DPTTF ligands (each DPTTF has four S atoms), meaning that approximately half of the DPTTF ligands were partially oxidized to DPTTF^•+^ radical cations, which were accompanied by an equal number of I_3_^−^ counterions for charge balance. Furthermore, based on the empirical formula and quantitative EPR analysis, we estimated that the pristine *sine*-MOF possessed 6.8 x 10^13^ spins/mg or 7.6 x 10^19^ spins/mol, which corresponded to only 0.01% DPTTF^•+^ population (possibly produced by negligible aerobic oxidation). In contrast, the I_2_-treated *sine*-MOF possessed 2.6 x 10^16^ spins/mg or 3.6 x 10^22^ spins/mol, which corresponded to a noticeably higher 6.1% DPTTF^•+^ population. Thus, elemental analysis and quantitative EPR analysis together helped us quantify the DPTTF^•+^ population in the I_2_-treated partially oxidized *sine*-MOF.

### Conductivity Measurements of Pristine and I_2_-Treated *sine*-MOFs

**FIGURE 7:**
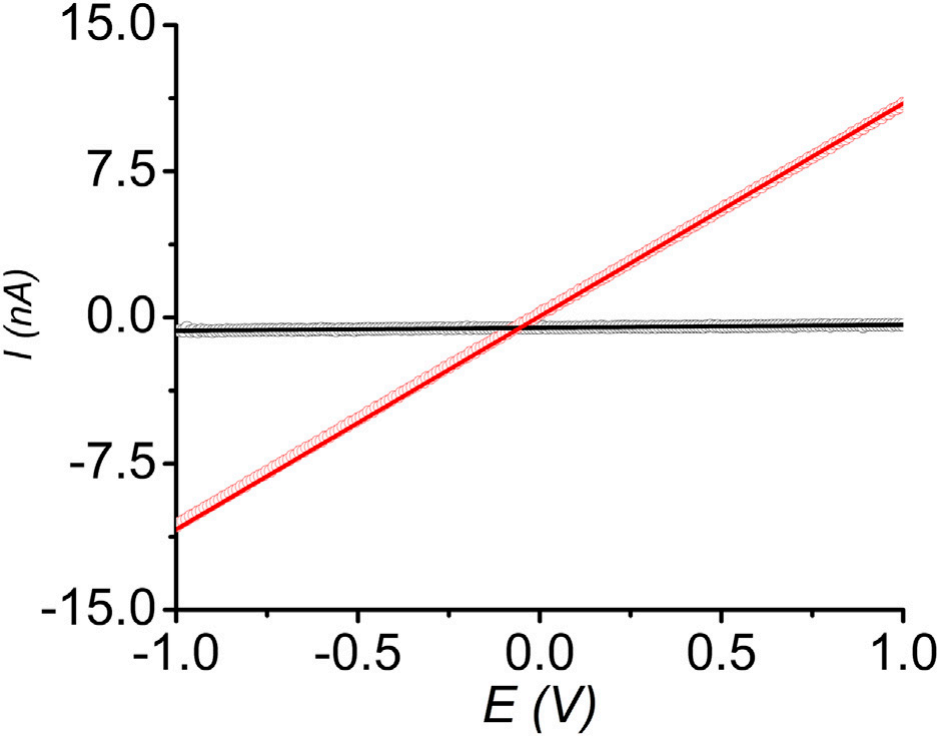
Linear *I-V* relationships of pristine (black) and I_2_-treated (red) *sine*-MOF measured by two-probe method.

Finally, we measured the room temperature electrical conductivity of pressed pellets of pristine and I_2_-treated *sine*-MOFs, which provided us insights into the effect of partial oxidation of *Z*-DPTTF ligands in the latter. DC-sweep measurements of pressed *sine*-MOF-pellets sandwiched between two conductive stainless-steel electrodes coated with Ag paste were conducted. Both materials displayed linear current-voltage (*I-V*) responses between –1 and +1 V (Figure 7), confirming ohmic contact between the pellets and electrodes. From the slopes of the corresponding *I-V* curves, the electrical conductivity of pristine and I_2_-treated *sine*-MOFs was determined to be 1 × 10^−8^ and 5 × 10^−7^ S/m, respectively. The 50-fold higher conductivity of I_2_-treated *sine*-MOF was attributed to partial oxidation of *Z*-DPTTF ligands to *Z*-DPTTF^•+^, which enhanced the charge carrier concentration. However, the increase was modest and the conductivity was still lower than other I_2_-treated TTF-based MOFs possibly because *sine*-MOF lacked sufficient π–π or S•••S interactions between the *Z*-DPTTF ligands, which hindered through-space charge movement, while the Zn_2_ paddlewheel nodes were not conducive to through-bond charge movement. As result, pristine and I_2_-treated *sine*-MOFs likely relied on less effective charge hopping mechanism, which caused modest conductivities even though the latter possessed larger number of mobile charge carriers due to the presence of DPTTF^•+^ radical cations.

## Conclusion

We have developed a new 3D *sine*-MOF structure featuring twisted TCPB ligands that formed Zn_2_(COO)_4_ paddlewheel nodes and axially coordinated U-shaped *Z*-DPTTF ligands that connected the adjacent nodes like sine-curves propagating along the b-axis. While the pristine *sine*-MOF displayed a modest intrinsic conductivity, its conductivity surged 50-fold to 5 × 10^−7^ S/m after iodine mediated partial oxidation of the electron rich *Z*-DPTTF ligands possibly due to enhanced charge carrier concentration. However, the lack of strong π–π- and S•••S interactions between the *Z*-DPTTF ligands hindered through-space charge movement, which was largely reliant on charge hopping, causing modest electrical conductivity of both pristine and I_2_-treated *sine*-MOFs. These studies not only presented a novel electronic MOF architecture, but also demonstrated that a high charge carrier concentration alone is not sufficient for high electrical conductivity unless a framework is also equipped with effective charge transport pathways.

## Data Availability

The *CIF* of the *sine*-MOF can be found in the Cambridge structural database with deposition number 2101676. The CIF and all the other additional data can also be found in the Supplementary Material.
