# Supplementary material for: A New Electrically Conducting Metal–Organic Framework Featuring U-Shaped cis-Dipyridyl Tetrathiafulvalene Ligands
Source: Front Chem. 2021 Oct 1;9:726544. doi: 10.3389/fchem.2021.726544 (PMC8517321; doi:10.3389/fchem.2021.726544)
Supplement: Supplementary file 1 [file DataSheet1.docx]

Supplementary Material

# Experimental Section

## General Materials and Methods

Reagents, starting materials and solvents were purchased from Sigma-Aldrich, Acros Organic, TCI America and used as received. Ag-paint was procured from Ted Pella. The electrodes (Ag/AgCl, Pt-mesh, Pt-disk, and glassy-carbon disc) and electrochemical cells were procured from BASi.

The single crystal X-ray diffraction (SXRD) data were collected on a Bruker D8 Venture dual source diffractometer equipped with Cu and Mo radiation sources and CMOS detector. The *sine-*MOF structure was solved and refined by using Bruker SHELXTL software package. The powder X-ray diffraction (PXRD) pattern of pristine and I_2_-treated sine-MOF powder was recorded on a Rigaku Ultima IV X-ray diffractometer equipped with Cu Kα radiation source (λ = 1.5406 Å) and a CCD area detector and Bruker D8 Venture dual source diffractometer.

The thermogravimetric analysis (TGA) was conducted under a N_2_ atmosphere using an SDT Q600 instrument.

Shimadzu UV-2600 spectrophotometer equipped with an integrating sphere (200–1400 nm range) was used to measure the diffuse-reflectance spectra of the pristine and iodine-treated MOFs and the absorption spectra of DPTTF ligand. The optical band-gaps were determined from the Tauc plots.

The electrochemical measurements of pristine and iodine-treated *sine-*MOF were conducted on a Princeton Applied Research VersaStat 3-450 instrument using a glassy carbon working electrode, Ag/AgCl reference electrode, Pt-mesh counter electrode, and a 0.1 M Bu_4_NPF_6_ in MeCN supporting electrolyte solution. The pastes of pristine and iodine-treated *sine-*MOFs made in MeCN were mounted onto the glassy carbon electrode surface to record their solid-state cyclic voltammograms.

The solid-state EPR spectra of pristine and iodine-treated *sine-*MOFs were recorded on a Bruker EMX EPR X-band spectrometer at room temperature.

The direct current-voltage (*I-V*) relationships of pristine and iodine-treated sine-MOF pellets sandwiched between two Ag-coated stainless steel electrodes (see below for pellet preparation) were recorded under ambient conditions between –1 to +1 V using a Keithley 2400 sourcemeter. The resistance of each device was extracted from the slope of the linear *I-V* plot and the corresponding conductivity was calculated using the equation σ = L/RA.; where σ = conductivity, L = thickness of the pellet, R = resistance of the pellet, and A = area of the pellet (πr2 , r = pellet radius = 1.35 mm), according to literature reports( Sun et al., 2016; Gordillo et al., 2020).

## MOF Pellets for Electrochemical Impedance and Electrical Conductivity Measurements.

To prepare pristine and iodine-treated MOF pellets for *dc*-sweep and *ac* impedance measurements, 3.0 mg of respective material was placed inside a Teflon tube (inner diameter 2.7 mm) capped on one side with a snugly fit stainless-steel rod (diameter 2.7 mm) with silver-coated tip. Then the other end of the Teflon tube was capped with another identical stainless-steel rod with silver-coated tip. Finally, the entire setup containing the MOF material, sandwiched between the two rods, was pressed under 200 MPa pressure using a digital Parr Pellet Press. The thickness of the resulting pellets (ca. 0.2 mm) was measured by a digital caliper from the difference in total length of the two steel rods with and without the sandwiched materials.

# Supplementary Figures and tables


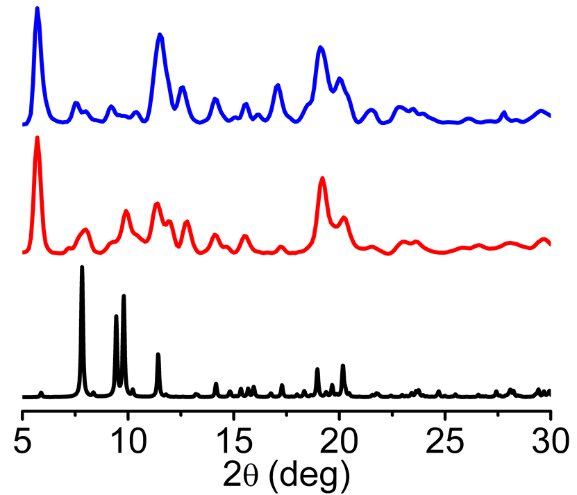


**Figure S1**. PXRD patterns of *sine-*MOF: simulated (black), pristine (red), and iodine-treated (blue).


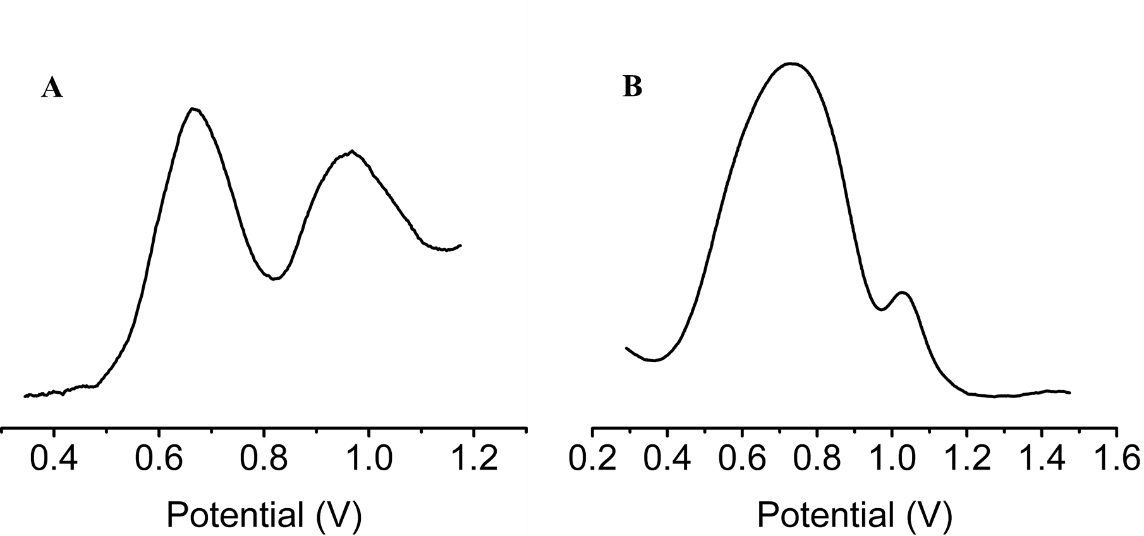


**Figure S2**. Square Wave Voltammetry (SWV) of A) pristine and B) I_2_-doped *sine-*MOF (vs Ag/AgCl, 0.1 M Bu_4_NPF_6_ in MeCN) showing the two stepwise one electron oxidation processes of DPTTF to DPTTF^•+^ and DPTTF^2+^.

**Table S1**. List of PXRD peaks of pristine and I_2_-doped *sine-*MOF

| **Calculated PRXD sine-MOF** | | | **Experimental PXRD sine-MOF** | | | **Taken in SXRD sine-MOF** | | | **Taken in SXRD I_2_-treated sine-MOF** | | |
| --- | --- | --- | --- | --- | --- | --- | --- | --- | --- | --- | --- |
| **2θ** | **Rel. Int.** | ***h k l*** | **2θ** | **Rel. Int.** | ***h k l*** | **2θ** | **Rel. Int.** | ***h k l*** | **2θ** | **Rel. Int.** | ***h k l*** |
| 5.88 | 3.45 | 0 2 0 | 7.82 | 38.4 | 2 0 0 | 5.7 | 100 | 0 2 0 | 5.7 | 100 | 0 2 0 |
| 7.82 | 100 | 2 0 0 | 8.06 | 41.95 | 2 0 0 | 8.0 | 21.1 | 2 0 0 | 7.5 | 16.7 | 2 0 0 |
| 8.36 | 3.67 | 2 1 0 | 8.52 | 17.66 | 2 1 0 | 9.2 | 9.6 | 0 1 1 | 8 | 9.5 | 2 0 0 |
| 9.44 | 62.2 | 0 1 1 | 9.50 | 100 | 0 1 1 | 9.9 | 37 | 2 2 0 | 9.2 | 13.7 | 0 1 1 |
| 9.80 | 77.8 | 2 2 0 | 9.84 | 47.3 | 2 2 0 | 11.4 | 43.2 | 1 2 1 | 10.4 | 8.7 | 1 1 1 |
| 10.22 | 6.02 | 1 1 1 | 11.24 | 47.4 | 1 2 1 | 11.9 | 28.1 | 2 0 1 | 11.5 | 76.9 | 1 2 1 |
| 11.42 | 33 | 1 2 1 | 11.52 | 43.7 | 1 2 1 | 12.8 | 27.9 | 3 2 0 | 12.6 | 30.4 | 0 3 1 |
| 11.80 | 2.1 | 0 4 0 | 12.00 | 22.9 | 2 0 1 | 14.1 | 16.6 | 2 4 0 | 14.1 | 20.4 | 2 4 0 |
| 13.28 | 2 | 1 3 1 | 12.66 | 35.4 | 0 3 1 | 14.7 | 6 | 0 5 0 | 15.6 | 15.9 | 4 0 0 |
| 14.16 | 10.37 | 2 4 0 | 12.88 | 70.1 | 3 2 0 | 15.5 | 15.5 | 1 4 1 | 16.2 | 6.4 | 4 1 0 |
| 14.82 | 4.38 | 0 4 1 | 14.94 | 17.9 | 2 3 1 | 17.2 | 6.1 | 3 3 1 | 17.1 | 32.4 | 3 3 1 |
| 15.36 | 5.74 | 1 4 1 | 15.44 | 23.6 | 1 4 1 | 19.2 | 65.2 | 4 2 1 | 19.1 | 65.6 | 4 2 1 |
| 15.68 | 6.7 | 4 0 0 | 15.74 | 18.09 | 4 0 0 | 20.2 | 31.5 | 4 3 1 | 20 | 38.2 | 4 3 1 |
| 15.94 | 7.9 | 4 1 0 | 16.00 | 51.2 | 4 1 0 | 21.5 | 6.1 | 0 4 2 | 21.5 | 10.7 | 0 4 2 |
| 16.76 | 2.96 | 4 2 0 | 16.46 | 19.2 | 3 4 0 | 23 | 10.4 | 5 4 0 | 22.8 | 13.2 | 2 4 2 |
| 17.28 | 9.2 | 0 5 1 | 17.18 | 95.9 | 3 3 1 | 23.6 | 10.6 | 6 0 0 | 23.5 | 11.6 | 5 3 1 |
| 18.00 | 2.14 | 0 0 2 | 17.90 | 55.3 | 0 0 2 | 25.8 | 5.3 | 1 8 1 | 23.9 | 7.4 | 4 0 2 |
| 18.34 | 4.74 | 4 1 1 | 19.12 | 58.9 | 4 2 1 | 26.6 | 7.6 | 5 6 0 | 26.1 | 3 | 4 7 0 |
| 18.66 | 1.73 | 1 1 2 | 19.34 | 57.2 | 2 6 0 | 28.1 | 9.4 | 5 6 1 | 27.8 | 8.5 | 0 2 3 |
| 18.96 | 21.49 | 3 4 1 | 19.74 | 66.3 | 4 4 0 | 29.7 | 12.7 | 6 6 0 | 28.4 | 1.7 | 2 1 3 |
| 19.38 | 3.97 | 2 6 0 | 20.14 | 75.4 | 4 3 1 |  |  |  | 29.5 | 9.8 | 6 5 1 |
| 19.66 | 9.38 | 4 4 0 | 20.44 | 33.2 | 1 3 2 |  |  |  |  |  |  |
| 19.88 | 2.51 | 5 1 0 | 21.44 | 46.2 | 2 6 1 |  |  |  |  |  |  |
| 20.16 | 24.26 | 4 3 1 | 21.62 | 65.3 | 4 5 0 |  |  |  |  |  |  |
| 20.46 | 3.2 | 1 3 2 | 22.14 | 24.4 | 2 7 0 |  |  |  |  |  |  |
| 21.56 | 1.82 | 0 4 2 | 22.84 | 34.5 | 2 4 2 |  |  |  |  |  |  |
| 21.76 | 3 | 3 1 2 | 23.46 | 38.7 | 5 3 1 |  |  |  |  |  |  |
| 22.44 | 1.23 | 5 2 1 | 23.94 | 32.2 | 4 0 2 |  |  |  |  |  |  |
| 22.98 | 1.7 | 2 4 2 | 24.66 | 27.5 | 4 2 2 |  |  |  |  |  |  |
| 23.42 | 3.3 | 5 3 1 | 25.36 | 27.7 | 0 8 1 |  |  |  |  |  |  |
| 23.62 | 4.85 | 6 0 0 | 27.02 | 56.7 | 1 9 0 |  |  |  |  |  |  |
| 23.74 | 5.42 | 0 8 0 | 27.34 | 99 | 1 0 3 |  |  |  |  |  |  |
| 23.96 | 1.63 | 4 0 2 | 28.00 | 56.5 | 5 6 1 |  |  |  |  |  |  |
| 24.70 | 4.45 | 4 2 2 | 29.24 | 64.8 | 3 9 0 |  |  |  |  |  |  |
| 24.98 | 1.07 | 2 8 0 | 29.56 | 59.7 | 3 0 3 |  |  |  |  |  |  |
| 25.48 | 2.13 | 6 1 1 | 29.70 | 45.6 | 6 6 0 |  |  |  |  |  |  |
| 26.58 | 1.85 | 5 6 0 | 29.82 | 32.1 | 3 1 3 |  |  |  |  |  |  |
| 26.95 | 1.9 | 1 9 0 |  |  |  |  |  |  |  |  |  |
| 27.42 | 4.5 | 1 0 3 |  |  |  |  |  |  |  |  |  |
| 28.08 | 5.8 | 5 6 1 |  |  |  |  |  |  |  |  |  |
| 28.24 | 4.95 | 5 3 2 |  |  |  |  |  |  |  |  |  |
| 29.30 | 3.2 | 3 9 0 |  |  |  |  |  |  |  |  |  |
| 29.42 | 5.99 | 6 5 1 |  |  |  |  |  |  |  |  |  |
| 29.68 | 4.49 | 6 6 0 |  |  |  |  |  |  |  |  |  |
| 29.94 | 4.94 | 1 4 3 |  |  |  |  |  |  |  |  |  |

**Table S2**. Conductivities of some TTF-based MOFs.

| **MOF Formula** | **Conductivity (S/m)** | **Methods** | **Reference** |
| --- | --- | --- | --- |
| Cd_2_(TTFTB) | 2.86 × 10^–2^ | Single crystal conductivity (Two Probe) | Park et al. 2015 |
| Zn_2_(TTFTB) | 3.95 × 10^–4^  2.5(2) × 10^−8^  1.6(2) × 10^−7^  (I_2_ dopped) | Single crystal conductivity (Two Probe)  Pellet | Park et al. 2015  Leong et al. 2018 |
| Mn_2_(TTFTB) | 8.64 × 10^–3^ | Single crystal conductivity (Two Probe) | Park et al. 2015 |
| Co_2_(TTFTB) | 1.49 × 10^–3^ | Single crystal conductivity (Two Probe) | Park et al. 2015 |
| {[Fe(dca)_2_][TTF(py)_4_]_0.5_  ⋅0.5 CH_2_Cl_2_]}*_n_* | 4.1×10^−7^  1.3×10^−4^  (I_2_ doped) | Pellet | Wang et al. 2017 |
| {[Fe(dca)][TTF(py)_4_]⋅ClO_4_  ⋅CH_2_Cl_2_⋅2 CH_3_OH]}*_n_* | 1.2×10^−5^  7.6×10^−3^ (I_2_ doped) | Pellet | Wang et al. 2017 |
| In(Me_2_NH_2_)(TTFTB) **1** | 1.23 × 10^−7^  5.50 × 10^−6^ (I_2_ doped) | Non specified | Su et al. 2017 |
| In(Me_2_NH_2_)(TTFTB) **2** | 1.16 × 10^−7^  1.68 × 10^−5^ (I_2_ doped) | Non specified | Su et al. 2017 |
| Zn_3_(ExTTFTB)_2_(H_2_O)_4_·6EtOH | 3.02 × 10^−8^  3.18 × 10^−4^  (I_2_ doped) | Pellet | Gordillo et al. 2020 |
| Zn_2_(DPTTF)(TCPB)·3DMA | 1 × 10^–8^  5 × 10^–7^ (I_2_ doped) | Pellet | This work |

# References

Leong, C. F., Wang, C.-H., Ling, C. D., and D’Alessandro, D. M. (2018). A spectroscopic and electrochemical investigation of a tetrathiafulvalene series of metal–organic frameworks. Polyhedron 154, 334–342. doi:10.1016/J.POLY.2018.07.023.

Gordillo, M. A.; Benavides, P. A.; Panda, D. K.; Saha, S. (2020) The Advent of Electrically Conducting Double-Helical Metal–Organic Frameworks Featuring Butterfly-Shaped Electron Rich π-Extended Tetrathiafulvalene Ligands. *ACS Appl. Mater. Interfaces* 12, 12955–12961.

Park, S. S., Hontz, E. R., Sun, L., Hendon, C. H., Walsh, A., Van Voorhis, T., et al. (2015). Cation-Dependent Intrinsic Electrical Conductivity in Isostructural Tetrathiafulvalene-Based Microporous Metal–Organic Frameworks. *J. Am. Chem. Soc.* 137, 1774–1777. doi:10.1021/ja512437u.

Su, J., Yuan, S., Wang, H.-Y., Huang, L., Ge, J.-Y., Joseph, E., et al. (2017). Redox-switchable breathing behavior in tetrathiafulvalene-based metal–organic frameworks. *Nat. Commun.* 8, 2008. doi:10.1038/s41467-017-02256-y.

Sun, L.; Park, S. S.; Sheberla, D.; Dinca, M. (2016). Measuring and Reporting Electrical Conductivity of in Metal-Organic Frameworks: Cd_2_(TTFTB) as a case study. *J. Am. Chem. Soc.* 138, 14772–14782.

Wang, H.-Y., Ge, J.-Y., Hua, C., Jiao, C.-Q., Wu, Y., Leong, C. F., et al. (2017). Photo- and Electronically Switchable Spin-Crossover Iron(II) Metal-Organic Frameworks Based on a Tetrathiafulvalene Ligand. *Angew. Chemie Int. Ed.* 56, 5465–5470. doi:10.1002/anie.201611824.

# Crystal Structure Report for [Zn_2_(DPTTF)TCPB•3DMA]_n_

The integration of the data using an orthorhombic unit cell yielded a total of 47751 reflections to a maximum θ angle of 25.25° (0.83 Å resolution), of which 6153 were independent (average redundancy 7.761, completeness = 99.9%, R_int_ = 6.60%, R_sig_ = 3.30%) and 5086 (82.66%) were greater than 2σ(F^2^). The final cell constants of a = 22.5570(7) Å, b = 30.0291(12) Å, c = 9.8469(4) Å, volume = 6670.0(4) Å^3^, are based upon the refinement of the XYZ-centroids of reflections above 20 σ(I). The calculated minimum and maximum transmission coefficients (based on crystal size) are 0.9321 and 1.0000. The structure was solved and refined using the Bruker SHELXTL Software Package, using the space group P n m a, with Z = 4 for the formula unit, C_62_H_55_N_5_O_11_S_4_Zn_2_. The final anisotropic full-matrix least-squares refinement on F^2^ with 482 variables converged at R1 = 6.94%, for the observed data and wR2 = 19.52% for all data. The goodness-of-fit was 1.087. The largest peak in the final difference electron density synthesis was 1.549 e^-^/Å^3^ and the largest hole was -1.327 e^-^/Å^3^ with an RMS deviation of 0.127 e^-^/Å^3^. On the basis of the final model, the calculated density was 1.300 g/cm^3^ and F(000), 2696 e^-^.

| **Table S3. Sample and crystal data** |
| --- |

| **Identification code** | D8_4427_B2_67 | |
| --- | --- | --- |
| **Chemical formula** | C_62_H_55_N_5_O_11_S_4_Zn_2_ | |
| **Formula weight** | 1305.09 g/mol | |
| **Temperature** | 100(2) K | |
| **Wavelength** | 0.71073 Å | |
| **Crystal size** | 0.050 x 0.110 x 0.210 mm | |
| **Crystal system** | orthorhombic | |
| **Space group** | P n m a | |
| **Unit cell dimensions** | a = 22.5570(7) Å | α = 90° |
|  | b = 30.0291(12) Å | β = 90° |
|  | c = 9.8469(4) Å | γ = 90° |
| **Volume** | 6670.0(4) Å^3^ |  |
| **Z** | 4 | |
| **Density (calculated)** | 1.300 g/cm^3^ | |
| **Absorption coefficient** | 0.903 mm^-1^ | |
| **F(000)** | 2696 | |

| **Table S4. Data collection and structure refinement** |
| --- |

| **Theta range for data collection** | 2.18 to 25.25° | |
| --- | --- | --- |
| **Index ranges** | -27<=h<=27, -35<=k<=36, -11<=l<=11 | |
| **Reflections collected** | 47751 | |
| **Independent reflections** | 6153 [R(int) = 0.0660] | |
| **Max. and min. transmission** | 1.0000 and 0.9321 | |
| **Structure solution technique** | direct methods | |
| **Structure solution program** | SHELXT 2014/5 (Sheldrick, 2014) | |
| **Refinement method** | Full-matrix least-squares on F^2^ | |
| **Refinement program** | SHELXL-2016/6 (Sheldrick, 2016) | |
| **Function minimized** | Σ w(F_o_^2^ - F_c_^2^)^2^ | |
| **Data / restraints / parameters** | 6153 / 191 / 482 | |
| **Goodness-of-fit on F^2^** | 1.087 | |
| **Δ/σ_max_** | 0.001 | |
| **Final R indices** | 5086 data; I>2σ(I) | R1 = 0.0694, wR2 = 0.1800 |
|  | all data | R1 = 0.0844, wR2 = 0.1952 |
| **Weighting scheme** | w=1/[σ^2^(F_o_^2^)+(0.0729P)^2^+51.1462P] where P=(F_o_^2^+2F_c_^2^)/3 | |
| **Largest diff. peak and hole** | 1.549 and -1.327 eÅ^-3^ | |
| **R.M.S. deviation from mean** | 0.127 eÅ^-3^ | |

| **Table S5. Atomic coordinates and equivalent isotropic atomic displacement parameters (Å^2^)** |
| --- |
| U(eq) is defined as one third of the trace of the orthogonalized U_ij_ tensor. |
|  |

|  | **x/a** | **y/b** | **z/c** | **U(eq)** |
| --- | --- | --- | --- | --- |
| Zn1 | 0.50424(2) | 0.48448(2) | 0.85836(6) | 0.01495(19) |
| S1 | 0.48591(7) | 0.30625(5) | 0.14365(14) | 0.0292(3) |
| S2 | 0.55094(6) | 0.30147(5) | 0.40395(14) | 0.0273(3) |
| O1 | 0.44516(17) | 0.53221(12) | 0.8057(4) | 0.0290(9) |
| O2 | 0.44083(17) | 0.55388(12) | 0.0225(4) | 0.0299(9) |
| O3 | 0.07036(17) | 0.53019(13) | 0.6581(4) | 0.0312(9) |
| O4 | 0.06523(17) | 0.55046(12) | 0.4401(4) | 0.0313(9) |
| N1 | 0.50957(19) | 0.44702(15) | 0.6878(5) | 0.0231(9) |
| C3 | 0.5110(2) | 0.3854(2) | 0.4763(6) | 0.0291(12) |
| C1 | 0.5553(4) | 0.4208(4) | 0.6685(10) | 0.031(2) |
| C2 | 0.5572(5) | 0.3897(4) | 0.5664(10) | 0.0319(19) |
| C4 | 0.4671(6) | 0.4183(4) | 0.4866(12) | 0.033(2) |
| C5 | 0.4679(6) | 0.4484(4) | 0.5923(13) | 0.031(2) |
| C1B | 0.5226(7) | 0.4014(5) | 0.7132(15) | 0.036(3) |
| C2B | 0.5257(7) | 0.3696(5) | 0.6119(15) | 0.034(2) |
| C4B | 0.4911(7) | 0.4252(5) | 0.4560(16) | 0.025(2) |
| C5B | 0.4910(7) | 0.4550(6) | 0.5642(17) | 0.026(3) |
| C6 | 0.5105(2) | 0.35078(19) | 0.3704(6) | 0.0270(12) |
| C7 | 0.4820(3) | 0.35225(19) | 0.2512(6) | 0.0296(12) |
| C8 | 0.5202(2) | 0.27239(18) | 0.2672(5) | 0.0238(11) |
| C9 | 0.4267(2) | 0.55734(16) | 0.8993(6) | 0.0223(11) |
| C10 | 0.3862(2) | 0.59473(17) | 0.8606(5) | 0.0200(10) |
| C11 | 0.3619(2) | 0.59683(16) | 0.7308(5) | 0.0205(11) |
| C12 | 0.3267(2) | 0.63296(16) | 0.6934(5) | 0.0200(10) |
| C13 | 0.3167(2) | 0.66768(16) | 0.7835(5) | 0.0193(10) |
| C14 | 0.3406(2) | 0.66521(17) | 0.9138(6) | 0.0231(11) |
| C15 | 0.3754(2) | 0.62881(17) | 0.9517(6) | 0.0241(11) |
| C16 | 0.2841(2) | 0.70881(15) | 0.7432(5) | 0.0166(10) |
| C17 | 0.2283(2) | 0.70923(16) | 0.6769(5) | 0.0177(10) |
| C18 | 0.2026(3) | 0.75 | 0.6439(7) | 0.0186(14) |
| C19 | 0.3104(3) | 0.75 | 0.7739(7) | 0.0169(14) |
| C20 | 0.0847(2) | 0.55416(17) | 0.5591(6) | 0.0225(11) |
| C21 | 0.1269(2) | 0.59193(16) | 0.5864(6) | 0.0208(11) |
| C22 | 0.1551(2) | 0.59600(17) | 0.7114(6) | 0.0236(11) |
| C23 | 0.1886(2) | 0.63335(16) | 0.7409(5) | 0.0207(10) |
| C24 | 0.1951(2) | 0.66726(16) | 0.6459(5) | 0.0176(10) |
| C25 | 0.1677(2) | 0.66292(16) | 0.5180(5) | 0.0211(11) |
| C26 | 0.1339(2) | 0.62557(17) | 0.4897(6) | 0.0233(11) |
| O27 | 0.1666(3) | 0.75 | 0.2964(7) | 0.0537(18) |
| N30 | 0.1436(4) | 0.75 | 0.0758(9) | 0.061(3) |
| C28 | 0.1287(5) | 0.75 | 0.2047(13) | 0.098(5) |
| C29 | 0.0655(8) | 0.75 | 0.238(2) | 0.161(7) |
| C31 | 0.1051(6) | 0.75 | 0.9590(13) | 0.113(7) |
| C32 | 0.2059(6) | 0.75 | 0.0439(16) | 0.143(10) |
| O33 | 0.3196(8) | 0.8054(6) | 0.3228(18) | 0.114(5) |
| N36 | 0.3827(8) | 0.7389(5) | 0.2401(16) | 0.094(4) |
| C34 | 0.3412(8) | 0.7651(5) | 0.2988(19) | 0.081(4) |
| C35 | 0.3117(10) | 0.7264(7) | 0.379(2) | 0.085(5) |
| C37 | 0.4115(11) | 0.7858(6) | 0.206(3) | 0.064(5) |
| C38 | 0.4084(11) | 0.6996(6) | 0.210(3) | 0.059(5) |
| O39 | 0.3049(6) | 0.8913(7) | 0.3238(17) | 0.119(5) |
| C41 | 0.2253(9) | 0.8831(10) | 0.162(2) | 0.118(6) |
| C40 | 0.2597(8) | 0.9074(8) | 0.269(2) | 0.124(5) |
| N42 | 0.2419(7) | 0.9489(8) | 0.319(2) | 0.125(5) |
| C43 | 0.2858(9) | 0.9711(9) | 0.402(2) | 0.115(6) |
| C44 | 0.1901(10) | 0.9664(11) | 0.239(3) | 0.143(7) |

| **Table S6. Bond lengths (Å)** |
| --- |

| Zn1-N1 | 2.024(5) | Zn1-O1 | 2.025(3) |
| --- | --- | --- | --- |
| Zn1-O3 | 2.033(4) | Zn1-O4 | 2.050(4) |
| Zn1-O2 | 2.059(4) | Zn1-Zn1 | 2.9474(11) |
| S1-C7 | 1.743(6) | S1-C8 | 1.764(5) |
| S2-C8 | 1.748(5) | S2-C6 | 1.771(6) |
| O1-C9 | 1.263(7) | O2-C9 | 1.259(7) |
| O3-C20 | 1.254(7) | O4-C20 | 1.256(7) |
| N1-C5B | 1.310(18) | N1-C1 | 1.312(11) |
| N1-C5 | 1.330(13) | N1-C1B | 1.424(15) |
| C3-C4B | 1.294(17) | C3-C2 | 1.376(11) |
| C3-C4 | 1.402(13) | C3-C2B | 1.455(16) |
| C3-C6 | 1.472(8) | C1-C2 | 1.374(14) |
| C1-H1 | 0.95 | C2-H2 | 0.95 |
| C4-C5 | 1.380(16) | C4-H4 | 0.95 |
| C5-H5 | 0.95 | C1B-C2B | 1.38(2) |
| C1B-H1B | 0.95 | C2B-H2B | 0.95 |
| C4B-C5B | 1.39(2) | C4B-H4B | 0.95 |
| C5B-H5B | 0.95 | C6-C7 | 1.339(8) |
| C7-H7 | 0.95 | C8-C8 | 1.345(11) |
| C9-C10 | 1.496(7) | C10-C15 | 1.382(7) |
| C10-C11 | 1.392(7) | C11-C12 | 1.394(7) |
| C11-H11 | 0.95 | C12-C13 | 1.387(7) |
| C12-H12 | 0.95 | C13-C14 | 1.393(7) |
| C13-C16 | 1.491(6) | C14-C15 | 1.397(7) |
| C14-H14 | 0.95 | C15-H15 | 0.95 |
| C16-C19 | 1.405(5) | C16-C17 | 1.418(7) |
| C17-C18 | 1.393(6) | C17-C24 | 1.498(6) |
| C18-H18 | 0.95 | C19-H19 | 0.95 |
| C20-C21 | 1.504(7) | C21-C22 | 1.392(7) |
| C21-C26 | 1.397(7) | C22-C23 | 1.383(7) |
| C22-H22 | 0.95 | C23-C24 | 1.391(7) |
| C23-H23 | 0.95 | C24-C25 | 1.409(7) |
| C25-C26 | 1.385(7) | C25-H25 | 0.95 |
| C26-H26 | 0.95 | O27-C28 | 1.244(14) |
| N30-C28 | 1.312(16) | N30-C32 | 1.440(16) |
| N30-C31 | 1.442(14) | C28-C29 | 1.46(2) |
| C29-H29A | 0.98 | C29-H29B | 0.98 |
| C29-H29C | 0.98 | C31-H31A | 0.98 |
| C31-H31B | 0.98 | C31-H31C | 0.98 |
| C32-H32A | 0.98 | C32-H32B | 0.98 |
| C32-H32C | 0.98 | O33-C34 | 1.326(16) |
| N36-C38 | 1.349(15) | N36-C34 | 1.352(15) |
| N36-C37 | 1.587(16) | C34-C35 | 1.555(16) |
| C35-H35A | 0.98 | C35-H35B | 0.98 |
| C35-H35C | 0.98 | C37-H37A | 0.98 |
| C37-H37B | 0.98 | C37-H37C | 0.98 |
| C38-H38A | 0.98 | C38-H38B | 0.98 |
| C38-H38C | 0.98 | O39-C40 | 1.250(16) |
| C41-C40 | 1.499(18) | C41-H41A | 0.98 |
| C41-H41B | 0.98 | C41-H41C | 0.98 |
| C40-N42 | 1.400(18) | N42-C43 | 1.449(18) |
| N42-C44 | 1.501(18) | C43-H43A | 0.98 |
| C43-H43B | 0.98 | C43-H43C | 0.98 |
| C44-H44A | 0.98 | C44-H44B | 0.98 |
| C44-H44C | 0.98 |  |  |

| **Table S7. Bond angles (°)** |
| --- |

| N1-Zn1-O1 | 102.70(17) | N1-Zn1-O3 | 105.40(18) |
| --- | --- | --- | --- |
| O1-Zn1-O3 | 89.11(17) | N1-Zn1-O4 | 94.98(17) |
| O1-Zn1-O4 | 87.69(16) | O3-Zn1-O4 | 159.58(17) |
| N1-Zn1-O2 | 97.23(16) | O1-Zn1-O2 | 159.76(16) |
| O3-Zn1-O2 | 88.94(16) | O4-Zn1-O2 | 87.13(17) |
| N1-Zn1-Zn1 | 164.68(13) | O1-Zn1-Zn1 | 88.62(12) |
| O3-Zn1-Zn1 | 84.80(12) | O4-Zn1-Zn1 | 74.97(12) |
| O2-Zn1-Zn1 | 71.14(11) | C7-S1-C8 | 93.4(3) |
| C8-S2-C6 | 94.0(3) | C9-O1-Zn1 | 117.0(3) |
| C9-O2-Zn1 | 138.4(4) | C20-O3-Zn1 | 121.0(3) |
| C20-O4-Zn1 | 132.7(4) | C1-N1-C5 | 118.1(8) |
| C5B-N1-C1B | 113.9(11) | C5B-N1-Zn1 | 130.6(8) |
| C1-N1-Zn1 | 120.0(5) | C5-N1-Zn1 | 121.8(6) |
| C1B-N1-Zn1 | 113.7(7) | C2-C3-C4 | 115.0(8) |
| C4B-C3-C2B | 121.5(10) | C4B-C3-C6 | 122.7(9) |
| C2-C3-C6 | 121.9(6) | C4-C3-C6 | 122.9(7) |
| C2B-C3-C6 | 114.9(7) | N1-C1-C2 | 122.6(9) |
| N1-C1-H1 | 118.7 | C2-C1-H1 | 118.7 |
| C1-C2-C3 | 120.8(9) | C1-C2-H2 | 119.6 |
| C3-C2-H2 | 119.6 | C5-C4-C3 | 120.5(10) |
| C5-C4-H4 | 119.8 | C3-C4-H4 | 119.8 |
| N1-C5-C4 | 121.5(10) | N1-C5-H5 | 119.2 |
| C4-C5-H5 | 119.2 | C2B-C1B-N1 | 123.3(13) |
| C2B-C1B-H1B | 118.4 | N1-C1B-H1B | 118.4 |
| C1B-C2B-C3 | 115.2(12) | C1B-C2B-H2B | 122.4 |
| C3-C2B-H2B | 122.4 | C3-C4B-C5B | 118.4(13) |
| C3-C4B-H4B | 120.8 | C5B-C4B-H4B | 120.8 |
| N1-C5B-C4B | 126.5(14) | N1-C5B-H5B | 116.7 |
| C4B-C5B-H5B | 116.7 | C7-C6-C3 | 127.0(5) |
| C7-C6-S2 | 116.0(4) | C3-C6-S2 | 117.0(4) |
| C6-C7-S1 | 118.9(5) | C6-C7-H7 | 120.6 |
| S1-C7-H7 | 120.6 | C8-C8-S2 | 119.97(18) |
| C8-C8-S1 | 125.19(18) | S2-C8-S1 | 114.7(3) |
| O2-C9-O1 | 124.8(5) | O2-C9-C10 | 117.5(5) |
| O1-C9-C10 | 117.7(5) | C15-C10-C11 | 119.5(5) |
| C15-C10-C9 | 119.9(5) | C11-C10-C9 | 120.5(5) |
| C10-C11-C12 | 120.1(5) | C10-C11-H11 | 119.9 |
| C12-C11-H11 | 119.9 | C13-C12-C11 | 120.5(5) |
| C13-C12-H12 | 119.7 | C11-C12-H12 | 119.7 |
| C12-C13-C14 | 119.1(5) | C12-C13-C16 | 122.1(4) |
| C14-C13-C16 | 118.7(4) | C13-C14-C15 | 120.3(5) |
| C13-C14-H14 | 119.8 | C15-C14-H14 | 119.8 |
| C10-C15-C14 | 120.3(5) | C10-C15-H15 | 119.8 |
| C14-C15-H15 | 119.8 | C19-C16-C17 | 117.8(4) |
| C19-C16-C13 | 117.6(4) | C17-C16-C13 | 124.6(4) |
| C18-C17-C16 | 119.0(4) | C18-C17-C24 | 118.9(4) |
| C16-C17-C24 | 122.0(4) | C17-C18-C17 | 122.9(6) |
| C17-C18-H18 | 118.5 | C17-C18-H18 | 118.5 |
| C16-C19-C16 | 123.4(6) | C16-C19-H19 | 118.3 |
| C16-C19-H19 | 118.3 | O3-C20-O4 | 125.7(5) |
| O3-C20-C21 | 117.2(5) | O4-C20-C21 | 117.0(5) |
| C22-C21-C26 | 119.2(4) | C22-C21-C20 | 120.9(5) |
| C26-C21-C20 | 119.7(5) | C23-C22-C21 | 120.5(5) |
| C23-C22-H22 | 119.8 | C21-C22-H22 | 119.8 |
| C22-C23-C24 | 120.7(5) | C22-C23-H23 | 119.7 |
| C24-C23-H23 | 119.7 | C23-C24-C25 | 119.2(4) |
| C23-C24-C17 | 122.1(4) | C25-C24-C17 | 118.7(4) |
| C26-C25-C24 | 119.8(5) | C26-C25-H25 | 120.1 |
| C24-C25-H25 | 120.1 | C25-C26-C21 | 120.7(5) |
| C25-C26-H26 | 119.6 | C21-C26-H26 | 119.6 |
| C28-N30-C32 | 117.5(11) | C28-N30-C31 | 128.1(10) |
| C32-N30-C31 | 114.5(11) | O27-C28-N30 | 121.7(11) |
| O27-C28-C29 | 120.6(13) | N30-C28-C29 | 117.7(13) |
| C28-C29-H29A | 109.5 | C28-C29-H29B | 109.5 |
| H29A-C29-H29B | 109.5 | C28-C29-H29C | 109.5 |
| H29A-C29-H29C | 109.5 | H29B-C29-H29C | 109.5 |
| N30-C31-H31A | 109.5 | N30-C31-H31B | 109.5 |
| H31A-C31-H31B | 109.5 | N30-C31-H31C | 109.5 |
| H31A-C31-H31C | 109.5 | H31B-C31-H31C | 109.5 |
| N30-C32-H32A | 109.5 | N30-C32-H32B | 109.5 |
| H32A-C32-H32B | 109.5 | N30-C32-H32C | 109.5 |
| H32A-C32-H32C | 109.5 | H32B-C32-H32C | 109.5 |
| C38-N36-C34 | 154.3(19) | C38-N36-C37 | 123.8(18) |
| C34-N36-C37 | 81.7(13) | O33-C34-N36 | 150.(2) |
| O33-C34-C35 | 116.(2) | N36-C34-C35 | 94.4(14) |
| C34-C35-H35A | 109.5 | C34-C35-H35B | 109.5 |
| H35A-C35-H35B | 109.5 | C34-C35-H35C | 109.5 |
| H35A-C35-H35C | 109.5 | H35B-C35-H35C | 109.5 |
| N36-C37-H37A | 109.5 | N36-C37-H37B | 109.5 |
| H37A-C37-H37B | 109.5 | N36-C37-H37C | 109.5 |
| H37A-C37-H37C | 109.5 | H37B-C37-H37C | 109.5 |
| N36-C38-H38A | 109.5 | N36-C38-H38B | 109.5 |
| H38A-C38-H38B | 109.5 | N36-C38-H38C | 109.5 |
| H38A-C38-H38C | 109.5 | H38B-C38-H38C | 109.5 |
| C40-C41-H41A | 109.5 | C40-C41-H41B | 109.5 |
| H41A-C41-H41B | 109.5 | C40-C41-H41C | 109.5 |
| H41A-C41-H41C | 109.5 | H41B-C41-H41C | 109.5 |
| O39-C40-N42 | 115.(2) | O39-C40-C41 | 123.(2) |
| N42-C40-C41 | 122.1(19) | C40-N42-C43 | 114.3(19) |
| C40-N42-C44 | 111.(2) | C43-N42-C44 | 132.(2) |
| N42-C43-H43A | 109.5 | N42-C43-H43B | 109.5 |
| H43A-C43-H43B | 109.5 | N42-C43-H43C | 109.5 |
| H43A-C43-H43C | 109.5 | H43B-C43-H43C | 109.5 |
| N42-C44-H44A | 109.5 | N42-C44-H44B | 109.5 |
| H44A-C44-H44B | 109.5 | N42-C44-H44C | 109.5 |
| H44A-C44-H44C | 109.5 | H44B-C44-H44C | 109.5 |

| **Table S8. Anisotropic atomic displacement parameters (Å^2^)** |
| --- |
| The anisotropic atomic displacement factor exponent takes the form: -2π^2^[ h^2^ a^*2^ U_11_ + ... + 2 h k a^*^ b^*^ U_12_ ] |

|  | **U_11_** | **U_22_** | **U_33_** | **U_23_** | **U_13_** | **U_12_** |
| --- | --- | --- | --- | --- | --- | --- |
| Zn1 | 0.0146(3) | 0.0096(3) | 0.0206(3) | 0.0030(2) | -0.0009(2) | 0.0001(2) |
| S1 | 0.0360(8) | 0.0310(8) | 0.0207(7) | 0.0003(6) | -0.0038(6) | 0.0035(6) |
| S2 | 0.0252(7) | 0.0289(7) | 0.0277(7) | -0.0032(6) | -0.0071(5) | 0.0036(5) |
| O1 | 0.032(2) | 0.025(2) | 0.030(2) | 0.0081(17) | 0.0037(17) | 0.0154(16) |
| O2 | 0.038(2) | 0.0178(19) | 0.034(2) | 0.0001(16) | -0.0169(18) | 0.0094(16) |
| O3 | 0.033(2) | 0.030(2) | 0.031(2) | -0.0066(18) | 0.0057(17) | -0.0171(17) |
| O4 | 0.032(2) | 0.022(2) | 0.040(2) | -0.0028(17) | -0.0141(18) | -0.0109(16) |
| N1 | 0.026(2) | 0.020(2) | 0.023(2) | 0.0001(19) | -0.0012(18) | 0.0058(18) |
| C3 | 0.027(2) | 0.032(3) | 0.028(3) | -0.004(2) | -0.002(2) | 0.004(2) |
| C1 | 0.026(4) | 0.039(5) | 0.028(4) | -0.009(4) | -0.007(3) | 0.006(4) |
| C2 | 0.030(4) | 0.035(4) | 0.031(4) | -0.006(4) | -0.003(3) | 0.010(3) |
| C4 | 0.035(5) | 0.031(4) | 0.031(5) | -0.006(4) | -0.007(4) | 0.011(4) |
| C5 | 0.032(6) | 0.027(5) | 0.033(5) | -0.005(4) | -0.008(5) | 0.011(5) |
| C1B | 0.041(5) | 0.034(5) | 0.031(5) | -0.004(5) | -0.010(5) | 0.011(5) |
| C2B | 0.040(5) | 0.031(5) | 0.033(5) | -0.004(4) | -0.007(4) | 0.011(4) |
| C4B | 0.024(5) | 0.027(5) | 0.026(5) | 0.004(4) | 0.004(5) | 0.004(5) |
| C5B | 0.025(6) | 0.026(5) | 0.027(6) | 0.006(5) | 0.005(5) | 0.004(5) |
| C6 | 0.023(3) | 0.030(3) | 0.028(3) | 0.002(2) | -0.001(2) | 0.003(2) |
| C7 | 0.032(3) | 0.030(3) | 0.026(3) | 0.003(2) | 0.001(2) | 0.003(2) |
| C8 | 0.020(2) | 0.032(3) | 0.019(3) | 0.000(2) | 0.000(2) | -0.001(2) |
| C9 | 0.017(2) | 0.014(2) | 0.036(3) | 0.007(2) | -0.003(2) | -0.0015(19) |
| C10 | 0.015(2) | 0.018(2) | 0.027(3) | 0.004(2) | -0.002(2) | 0.0017(19) |
| C11 | 0.023(2) | 0.013(2) | 0.026(3) | 0.002(2) | 0.000(2) | 0.0046(19) |
| C12 | 0.020(2) | 0.015(2) | 0.024(3) | 0.001(2) | -0.006(2) | 0.0028(19) |
| C13 | 0.015(2) | 0.017(2) | 0.027(3) | 0.002(2) | -0.004(2) | -0.0019(18) |
| C14 | 0.028(3) | 0.015(2) | 0.026(3) | -0.001(2) | -0.008(2) | 0.004(2) |
| C15 | 0.027(3) | 0.019(3) | 0.027(3) | 0.001(2) | -0.009(2) | 0.006(2) |
| C16 | 0.014(2) | 0.013(2) | 0.023(2) | -0.002(2) | -0.0033(19) | 0.0012(18) |
| C17 | 0.018(2) | 0.013(2) | 0.021(2) | -0.002(2) | -0.003(2) | -0.0012(19) |
| C18 | 0.014(3) | 0.017(3) | 0.024(4) | 0 | -0.008(3) | 0 |
| C19 | 0.012(3) | 0.011(3) | 0.027(4) | 0 | -0.009(3) | 0 |
| C20 | 0.016(2) | 0.015(3) | 0.037(3) | -0.011(2) | 0.000(2) | 0.0009(19) |
| C21 | 0.018(2) | 0.013(2) | 0.032(3) | -0.004(2) | -0.003(2) | -0.0034(19) |
| C22 | 0.024(3) | 0.018(3) | 0.029(3) | 0.002(2) | -0.004(2) | -0.002(2) |
| C23 | 0.018(2) | 0.018(2) | 0.026(3) | -0.001(2) | -0.005(2) | -0.0040(19) |
| C24 | 0.015(2) | 0.013(2) | 0.025(3) | -0.003(2) | -0.0055(19) | 0.0011(18) |
| C25 | 0.024(2) | 0.010(2) | 0.029(3) | 0.000(2) | -0.005(2) | -0.0029(19) |
| C26 | 0.020(2) | 0.020(3) | 0.030(3) | -0.005(2) | -0.008(2) | 0.002(2) |
| O27 | 0.061(4) | 0.059(5) | 0.041(4) | 0 | -0.017(4) | 0 |
| N30 | 0.046(5) | 0.097(8) | 0.041(5) | 0 | -0.006(4) | 0 |
| C28 | 0.039(6) | 0.215(17) | 0.041(7) | 0 | -0.003(5) | 0 |
| C29 | 0.082(10) | 0.32(2) | 0.085(11) | 0 | -0.003(9) | 0 |
| C31 | 0.042(7) | 0.25(2) | 0.045(7) | 0 | -0.014(6) | 0 |
| C32 | 0.047(8) | 0.32(3) | 0.062(9) | 0 | 0.011(7) | 0 |
| O33 | 0.130(11) | 0.118(11) | 0.093(10) | -0.019(9) | -0.035(9) | 0.008(10) |
| N36 | 0.115(7) | 0.099(9) | 0.067(6) | -0.007(6) | -0.041(6) | 0.013(6) |
| C34 | 0.092(7) | 0.098(9) | 0.054(6) | -0.006(6) | -0.031(6) | 0.008(6) |
| C35 | 0.087(11) | 0.111(12) | 0.057(10) | 0.016(9) | -0.004(9) | -0.006(10) |
| C37 | 0.078(7) | 0.058(9) | 0.058(7) | 0.004(7) | -0.028(6) | -0.011(7) |
| C38 | 0.072(7) | 0.051(8) | 0.053(7) | -0.011(7) | -0.024(6) | 0.032(7) |
| O39 | 0.047(7) | 0.204(14) | 0.106(10) | 0.044(10) | -0.021(7) | -0.018(8) |
| C41 | 0.049(9) | 0.211(17) | 0.095(12) | 0.012(13) | 0.006(9) | 0.008(11) |
| C40 | 0.052(7) | 0.204(13) | 0.114(9) | 0.049(9) | 0.025(7) | 0.011(8) |
| N42 | 0.061(7) | 0.197(13) | 0.119(9) | 0.055(9) | 0.036(7) | 0.018(8) |
| C43 | 0.062(10) | 0.190(16) | 0.093(12) | 0.073(12) | 0.008(9) | 0.021(11) |
| C44 | 0.087(11) | 0.206(17) | 0.137(14) | 0.062(14) | 0.054(11) | 0.035(12) |

| **Table S9. Hydrogen atomic coordinates and isotropic atomic displacement parameters (Å^2^)** |
| --- |

|  | **x/a** | **y/b** | **z/c** | **U(eq)** |
| --- | --- | --- | --- | --- |
| H1 | 0.5885 | 0.4235 | 0.7274 | 0.037 |
| H2 | 0.5909 | 0.3708 | 0.5580 | 0.038 |
| H4 | 0.4365 | 0.4198 | 0.4204 | 0.039 |
| H5 | 0.4381 | 0.4707 | 0.5969 | 0.037 |
| H1B | 0.5294 | 0.3923 | 0.8043 | 0.043 |
| H2B | 0.5367 | 0.3396 | 0.6295 | 0.041 |
| H4B | 0.4768 | 0.4340 | 0.3692 | 0.03 |
| H5B | 0.4761 | 0.4840 | 0.5466 | 0.031 |
| H7 | 0.4601 | 0.3779 | 0.2256 | 0.035 |
| H11 | 0.3694 | 0.5736 | 0.6676 | 0.025 |
| H12 | 0.3094 | 0.6338 | 0.6055 | 0.024 |
| H14 | 0.3331 | 0.6884 | 0.9771 | 0.028 |
| H15 | 0.3917 | 0.6275 | 1.0405 | 0.029 |
| H18 | 0.1658 | 0.7500 | 0.5969 | 0.022 |
| H19 | 0.3479 | 0.7500 | 0.8177 | 0.02 |
| H22 | 0.1514 | 0.5729 | 0.7770 | 0.028 |
| H23 | 0.2073 | 0.6359 | 0.8270 | 0.025 |
| H25 | 0.1724 | 0.6855 | 0.4514 | 0.025 |
| H26 | 0.1152 | 0.6228 | 0.4036 | 0.028 |
| H29A | 0.0423 | 0.7500 | 0.1535 | 0.241 |
| H29B | 0.0559 | 0.7234 | 0.2907 | 0.241 |
| H29C | 0.0559 | 0.7766 | 0.2907 | 0.241 |
| H31A | 0.1290 | 0.7500 | -0.1241 | 0.17 |
| H31B | 0.0800 | 0.7234 | -0.0392 | 0.17 |
| H31C | 0.0800 | 0.7766 | -0.0392 | 0.17 |
| H32A | 0.2111 | 0.7500 | -0.0549 | 0.214 |
| H32B | 0.2245 | 0.7766 | 0.0824 | 0.214 |
| H32C | 0.2245 | 0.7234 | 0.0824 | 0.214 |
| H35A | 0.3307 | 0.6982 | 1.3540 | 0.127 |
| H35B | 0.3164 | 0.7316 | 1.4765 | 0.127 |
| H35C | 0.2694 | 0.7251 | 1.3563 | 0.127 |
| H37A | 0.3850 | 0.8097 | 1.2371 | 0.097 |
| H37B | 0.4497 | 0.7885 | 1.2529 | 0.097 |
| H37C | 0.4175 | 0.7884 | 1.1081 | 0.097 |
| H38A | 0.4433 | 0.7047 | 1.1531 | 0.089 |
| H38B | 0.4206 | 0.6848 | 1.2946 | 0.089 |
| H38C | 0.3801 | 0.6807 | 1.1616 | 0.089 |
| H41A | 0.1917 | 0.9015 | 1.1324 | 0.178 |
| H41B | 0.2511 | 0.8771 | 1.0838 | 0.178 |
| H41C | 0.2105 | 0.8550 | 1.1987 | 0.178 |
| H43A | 0.2701 | 0.9998 | 1.4333 | 0.173 |
| H43B | 0.2951 | 0.9524 | 1.4811 | 0.173 |
| H43C | 0.3218 | 0.9760 | 1.3488 | 0.173 |
| H44A | 0.1782 | 0.9954 | 1.2754 | 0.214 |
| H44B | 0.2015 | 0.9696 | 1.1436 | 0.214 |
| H44C | 0.1569 | 0.9455 | 1.2463 | 0.214 |
